# Autoimmune Disease: Phthalate Linked to Lupus in Mice

**DOI:** 10.1289/ehp.113-a809a

**Published:** 2005-12

**Authors:** Carol Potera

No one knows to what degree genetics or environmental agents cause lupus, an autoimmune disorder that affects the skin, joints, and internal organs including the kidneys. However, researchers at Indiana State University may have strengthened the environmental evidence by discovering that phthalates trigger lupus antibodies in a mouse model.

Phthalates are found in adhesives, cosmetics, fragrances, vinyl flooring, polyvinyl chloride pipe, and certain toys and medical supplies. According to a report out of the Centers for Disease Control and Prevention and the National Toxicology Program, published in the October 2000 issue of *EHP*, phthalate exposure is more extensive than previously suspected, especially in women aged 20–40 years. Other studies have pointed to possible links with asthma, rhinitis, and eczema in children as well as altered genital development in male infants. The new lupus findings add to a growing list of potential health effects caused by these chemicals.

In lupus, the immune system loses its ability to tell the difference between foreign substances (antigens) and the body’s own cells and tissues. The immune system makes antibodies against the body itself, causing inflammation, tissue injury, and pain. Up to 1.5 million Americans have been diagnosed with lupus, and another 16,000 develop the disease each year, according to the Lupus Foundation of America.

While investigating the gene sequence of a monoclonal antibody used as a marker for tumor growth, biochemist Swapan Ghosh, interim chair of the Life Sciences Department at Indiana State University, noticed that it shared 98% similarity with an antibody protein component (light chain) made by NZB mice, a popular model for autoimmune diseases. In lupus, such antibodies attack DNA in the kidneys, heart, and lungs. The finding, published in the December 2003 issue of *Immunology*, was a surprise: “I was not studying lupus or autoimmune diseases at all,” says Ghosh. But he took advantage of the unexpected turn and has launched a series of experiments to further explore the phthalate–lupus connection.

In the latest study, Ghosh and graduate student So-Yon Lim injected four types of mice, including NZB mice, with di-(2-ethylhexyl) phthalate, or DEHP. Initially, all the mice generated antiphthalate antibodies, but only the lupus-prone NZB mice developed nephritis, which led to kidney failure and early death. The other mice initially produced antiphthalate antibodies, but the antibodies were counteracted by CD8+ suppressor T cells, which prevented kidney damage. “There’s something different about the immune systems of NZB mice [that makes them more susceptible to phthalates],” says Ghosh. The details of the investigation are reported in the August 2005 issue of the *Journal of Autoimmunity*.

Although the phthalate–lupus connection has been observed only in mice, “many things found in the mouse immune system have proven to be true in humans,” says Ghosh. On the other hand, “not everything seen in a mouse model reflects what happens in humans,” cautions Betty Diamond, chief of rheumatology at Columbia University.

Although Ghosh’s results are far from applicable to humans, they do raise several questions for future studies on the potential phthalate–lupus link in people. Do lupus patients have high levels of antiphthalate antibodies? Ghosh plans to screen lupus patients and healthy people in the future to find out. Does exposure to phthalates increase the risk for lupus? He plans to explore this, too, by measuring blood levels in workers exposed to phthalates in the plastics manufacturing industry. Lupus is five times more common in women than men. Might this be because women use more phthalate-containing cosmetics and perfumes than men do?

The American Chemistry Council (ACC), an industry trade group, has criticized Ghosh’s study because he combined DEHP to proteins like bovine serum albumin. “The attached proteins may cause autoimmune and allergic responses,” says Marian Stanley, director of the ACC’s Phthalate Esters Panel. Ghosh counters, “We also studied DEHP not complexed to a protein, and it evoked an anti-DNA response.” He explains that he attached a main metabolite of DEHP to proteins because some studies have suggested that phthalate metabolites show an affinity for albumin in the body.

So far, exposure to ultraviolet light is the only environmental factor that has been clearly linked to lupus in genetically susceptible patients. As lupus researchers continue to investigate other environmental causes, “we need to be open-minded, but not jump to conclusions,” Diamond says.

